# Growth Hormones in Broad Bean (*Vicia faba* L.) and Radish (*Raphanus raphanistrum* subsp. *sativus* L.) Are Associated with Accumulated Concentrations of Perfluoroalkyl Substances

**DOI:** 10.3390/toxics11110922

**Published:** 2023-11-11

**Authors:** Thimo Groffen, Niels Kuijper, Sevgi Oden, Tim Willems, Lieven Bervoets, Els Prinsen

**Affiliations:** 1 ECOSPHERE, Department of Biology, University of Antwerp, Groenenborgerlaan 171, 2020 Antwerp, Belgium; ng.kuijper@student.maastrichtuniversity.nl (N.K.); lieven.bervoets@uantwerpen.be (L.B.); 2 Integrated Molecular Plant Physiology Research (IMPRes), Department of Biology, University of Antwerp, Groenenborgerlaan 171, 2020 Antwerp, Belgium; sevgi.oden@uantwerpen.be (S.O.); tim.willems@uantwerpen.be (T.W.); els.prinsen@uantwerpen.be (E.P.)

**Keywords:** PFAS, phytotoxicity, plant hormones, gibberellins, methionine, IAA

## Abstract

In this study, we grew radish (*Raphanus raphanistrum* subsp. *sativus* L.) and broad beans (*Vicia faba* L.) in a greenhouse on soils spiked with a mixture of 15 per- and polyfluoroalkyl substances (PFASs) and investigated the association between accumulated ∑PFAS concentrations, growth, and hormone levels. Short-chained PFASs dominated aboveground tissues, whereas long-chained PFASs were most abundant in the plant roots. Our results showed that the presence or absence of exodermal Casparian strips, as well as the hydrophobicity and anion exchange capacities of PFASs, could explain the translocation of PFASs within plants. Significant associations found between accumulated PFAS concentrations and levels of gibberellins (GA1 and GA15), methionine, and indole-3-acetic acid (IAA) imply potential effects of PFASs on plant development and growth. This study provides the first evidence of associations between PFAS accumulation in plants and growth hormone levels, possibly leading to growth reduction of the apical dome and effects on the cell cycle in pericycle cells and methionine metabolism in plants.

## 1. Introduction

Per- and polyfluoroalkyl substances (PFASs) are chemicals that are widely used in industries and consumer applications due to their amphiphilic, chemical-, and thermal-resistant properties [1]. Decades-long disposal resulted in contamination of the environment [2,3]. PFASs tend to accumulate in the tissues of humans, wildlife, and plants [4,5], where they may cause severe toxic effects [5,6]. Despite several regulations for some long-chained PFASs, environmental concentrations are often still high [4,7]. Furthermore, the alternatives used, particularly short-chained PFASs, have higher mobility in the environment due to their higher water solubility and lower sorption of solids [8].

PFASs that are present in groundwater or porewater may be actively or passively taken up by plants. Subsequently, PFASs may be transported within plants following transpiration streams from belowground to aboveground tissues [9]. Here, PFASs may end up in edible tissues, from where they can contribute to human exposure [10]. PFAS uptake by plants is known to be both species-specific and compound-specific [4,11]. Nonetheless, previous studies on PFAS accumulation by plants have been primarily conducted on cereals such as maize, wheat, and oats. Many of these studies have focused particularly on perfluorooctane sulfonate (PFOS) and perfluorooctanoic acid (PFOA) (reviewed by Ghisi et al. [12]), but data for other plant species as well as other PFASs is still lacking.

Furthermore, the effects of many PFASs on plants are still poorly understood [6,13], and this information could be useful for ecological risk assessment. Although there are some studies that have reported the effects of PFAS exposure on plant growth (reviewed by Li et al. [13]), the potential mechanisms behind these effects have not been reported before. Plant hormones play a crucial role in controlling fundamental processes in plant growth and development [14] because they regulate the growth rate of individual parts and cooperate to modify biological responses for the formation and maintenance of plant stress tolerance [15]. Thus, any factor affecting plant hormones and plant growth will directly affect the fitness of the plant, resulting in both ecological and economic consequences. 

In this study, we determined the distribution of a selection of PFASs in different parts of cultivated vegetables (radish (*Raphanus raphanistrum* subsp. *sativus* L.) and broad beans (*Vicia faba* L.)) grown in spiked soils under greenhouse conditions, using environmentally relevant concentrations. Furthermore, associations between accumulated ∑PFAS concentrations and growth hormone levels were investigated.

## 2. Materials and Methods

### 2.1. Experimental Setup

Thirty plants of radish and broad bean were sown and grown for 8 weeks, from September 2021 until November 2021, in 10 × 10 cm plastic pots (one plant per pot) in a greenhouse setting (Wilrijk, Belgium: 51°09′19.4″ N, 4°24′29.1″ E). This allowed for growth under controlled conditions, with fluctuating temperatures, photoperiods, and relative humidity similar to that of the environment. The soil (peat potting medium (57% soil water content, Jiffy Products International B.V., Zwijndrecht, The Netherlands) of fifteen plants per species was spiked (by adding spiked PFAS-free tap water to the soil and thorough mixing by hand) with a PFAS mixture (PFAS standard M-537 Native Compound standard, Accustandard, New Haven, CT, USA), containing C6-C14 perfluoroalkyl carboxylic acids (PFCAs) and C4, C6, and C8 perfluoroalkyl sulfonic acids (PFSAs), and individual standards of perfluorobutanoic acid (PFBA), perfluoropentanoic acid (PFPeA), and perfluorobutane sulfonamide (FBSA) (Wellington Laboratories, Guelph, Canada), resulting in a final concentration of 7.64 ± 1.40 ng/g dry weight (dw) each (mean ± standard deviation) (Table S1). Although all compounds were added at the same concentration, the concentrations used were environmentally relevant as they were within the range of those observed at a fluorochemical hotspot in Antwerp [7]. The other fifteen plants per species were used as a control treatment and grew on uncontaminated (non-spiked) soil. The moisture content was determined gravimetrically, and PFAS-free tap water was added every two days to keep the moisture content constant. Every two days, growth and morphology were assessed, and pots were randomized. After eight weeks, the plants were harvested. Leaves, tubers, and roots of *R. raphanistrum* as well as upper leaves (above the flowers), lower leaves (below the flowers), flowers, apex, and stem of *V. faba* were collected separately into 50 mL polypropylene (PP) tubes (Sarstedt bv, Antwerp, Belgium) and flash frozen in liquid nitrogen. Afterwards, all samples were stored at −80 °C.

### 2.2. Sample Pre-Treatment and Extraction

Per matrix, five random samples were oven-dried to determine the dry weight in the plant matrices. An electric blender (Bamix, Mettlen, Switzerland) was used to homogenize the tubers. Homogenization of the other plant tissues occurred by grinding them to a fine powder using a mortar and pestle. At all times, samples were kept frozen (using liquid nitrogen) to prevent degradation of the plant hormones. Of every sample, 0.15 g was collected in 50 mL PP tubes for PFAS extraction, and another 0.15 g was stored in PP Eppendorf tubes for hormone extraction.

The PFASs were extracted from the samples following an adapted protocol by [16]. Details on the extraction method are provided in Appendix A. Briefly, samples were spiked with 10 ng of internal standards (MPFAC-MXA, Wellington Laboratories, Guelph, Canada), followed by a solvent extraction with acetonitrile (ACN). After extracting overnight, the extracts were cleaned up using graphitized activated carbon powder, followed by drying completely under vacuum. Hereafter, the samples were reconstituted with 2% ammonium hydroxide in ACN and filtered into PP auto-injector vials prior to UPLC-MS/MS analyses. 

A total of 5 exposed and 5 control samples from *V. faba* (apex and upper leaf tissues) and *R. raphanistrum* (tuber and leaf tissues) were selected for hormone analysis. Targeted hormone fractions included (1) the aminocyclopropane-1-carboxylic acid (ACC) and cytokinins fraction, (2) the total ACC fraction, (3) the indole-3-acetic acid (IAA) fraction, including gibberellins (GA), jasmonic acid (JA), and abscisic acid (ABA), and (4) the total IAA fraction. Furthermore, the amino acid methionine (MET) was targeted since it is involved in the biosynthesis of the plant hormone ethylene. The details on the extraction of the plant hormones are provided in Appendix A.

All samples were analyzed using ultra-performance liquid chromatography tandem mass spectrometry (UPLC-MS/MS), with different conditions depending on the targeted analytes. The instrumental settings are mentioned in Appendix A. Instrumental blanks (100% ACN) were injected at regular intervals to prevent cross-over PFAS contamination between injections. One procedural blank, consisting of 10 mL of ACN, was used per batch of 15 samples. This allowed for correction of contamination that might have occurred during the extraction and analysis. Contamination in these blanks were subtracted from concentrations in the samples of the same batch. The limit of quantification (LOQ) was determined for the individual PFASs in matrix (regardless of the specific plant tissue) as the concentration corresponding to a signal-to-noise ratio of 10 and is displayed in Table S2.

### 2.3. Statistical Analyses

The statistical analyses were performed in the statistical computing software R (version 4.1.3). The level of significance was set at *p* ≤ 0.05. The Shapiro–Wilk test was used to examine the validity of the model’s assumptions, and non-parametric alternatives were used in cases where normality assumptions were violated. The PFAS concentrations below the LOQ were replaced with a value of LOQ/2 [17]. The statistical analyses were performed on PFASs and hormones with a detection frequency of at least 66.7% and 80%, respectively. Differences in PFAS concentrations between controls and exposed plants were determined using a two-tailed, two-sample T-test or a two-sided, unpaired Wilcoxon test. Heatmaps were created to provide an overview of the different PFAS concentrations in the different plant compartments. The differences between these compartments were statistically compared using ANOVA, followed by a Tukey post hoc analysis. Accumulated PFAS concentrations were correlated between the different plant compartments using Spearman’s correlation tests. The same tests were used to correlate accumulated PFAS concentrations with growth hormone concentrations.

## 3. Results

### 3.1. Accumulated PFAS Concentrations and Profiles

**Figure 1:**
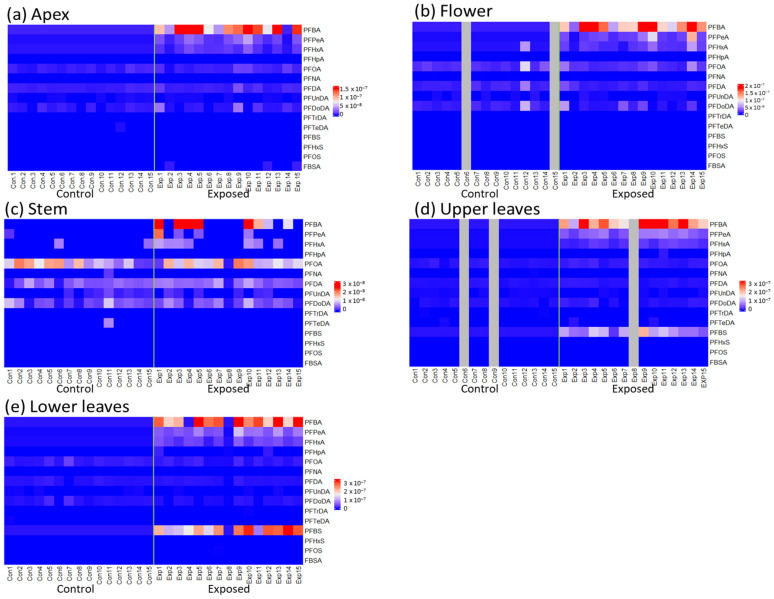
Heatmaps representing the relative PFAS concentrations in *V. faba* in mmol/g dw. The highest PFAS concentrations are shown in red, whereas the lowest concentrations are shown in blue. *n* = 15 for apex (**a**), stem (**c**), and lower leaves (**e**). *n* = 13 for control flowers, *n* = 15 for exposed flowers (**b**), *n* = 13 for control upper leaves, and *n* = 14 for exposed upper leaves (**d**). The green line in each heatmap separates the control (Con) and exposed (Exp) plants on the x-axis.

The concentrations of PFASs (PFBA, PFPeA, perfluorohexanoic acid (PFHxA), perfluoroheptanoic acid (PFHpA), PFOA, perfluorononanoic acid (PFNA), perfluorodecanoic acid (PFDA), perfluoroundecanoic acid (PFUnDA), perfluorododecanoic acid (PFDoDA), perfluorotridecanoic acid (PFTrDA), perfluorotetradecanoic acid (PFTeDA), perfluorobutane sulfonate (PFBS), perfluorohexane sulfonate (PFHxS), PFOS, and FBSA) in tissues collected from both control and exposed groups of *V. faba* ranged between <LOQ and 78.5 ng/g dw (Figure 1 and Table S4 for details on concentrations). When comparing PFAS concentrations within the same plant tissue, the PFAS concentrations were expressed in mmol/g dw, instead of ng/g dw, to correct for differences in molecular mass among the different PFASs.

**Figure 2:**
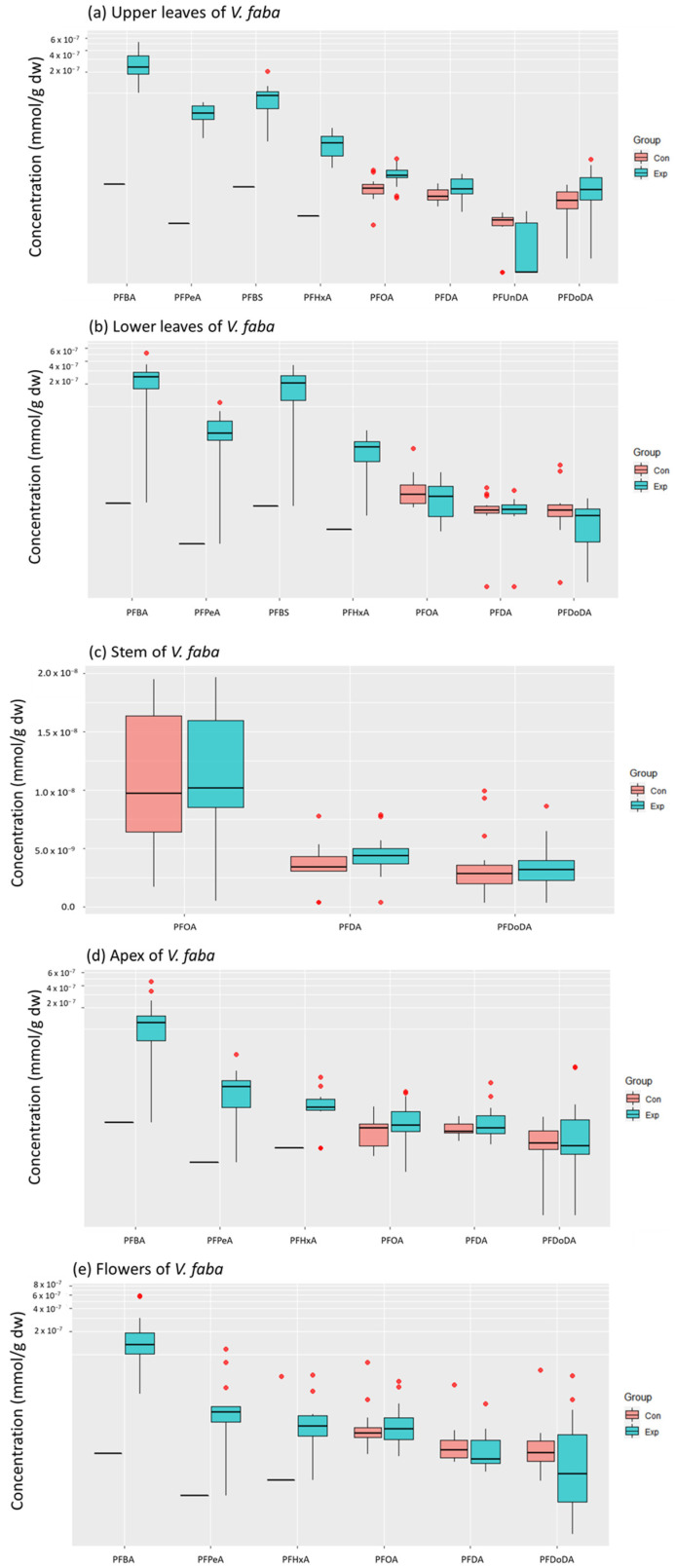
PFAS concentrations (mmol/g dw) in (**a**) upper leaves (*n* = 13 for control, *n* = 14 for exposed), (**b**) lower leaves (*n* = 15 per treatment), (**c**) stem (*n* = 15 per treatment), (**d**) apex (*n* = 15 per treatment), and (**e**) flowers (*n* = 13 for control, *n* = 15 for exposed) of exposed (Exp, blue) and control (Con, red) plants of *V. faba*. Outliers are indicated as red data points. Only compounds detected in at least 2/3 of the samples were included.

Concentrations of short-chained PFASs were highest in the leaves, apex, and flowers of *V. faba*, although there were some exceptions. Accumulated concentrations in the leaves tended to decrease with increasing carbon-chain length. The concentrations of short-chained PFBA, PFPeA, PFHxA, and PFBS were significantly higher in the leaves of the exposed plants compared to those of the controls (*p* < 0.05, Figure 2), whereas no significant differences were observed for the long-chained PFASs. Similar patterns were observed for the apex and flowers, although PFPeA concentrations in the apex and PFHxA concentrations in the flowers did not significantly differ between control and exposed plants (Figure 2). On the other hand, long-chained PFASs dominated the PFAS profiles and concentrations in the stem, although these concentrations did not differ significantly between exposed and control plants.

**Figure 3:**
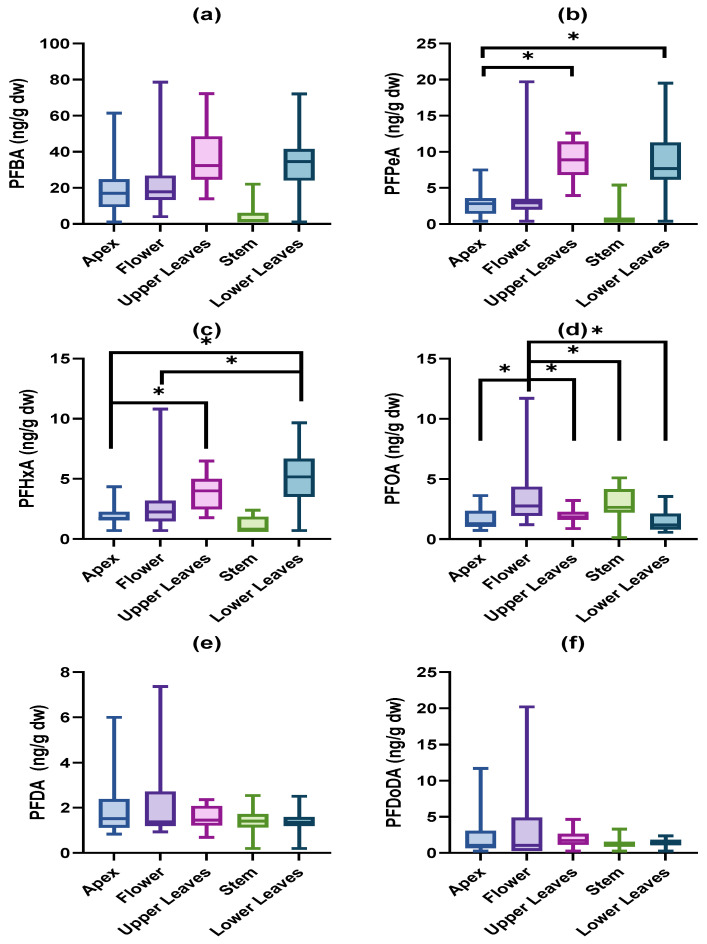
Concentrations (ng/g dw) of (**a**) PFBA, (**b**) PFPeA, (**c**) PFHxA, (**d**) PFOA, (**e**) PFDA, and (**f**) PFDoDA in exposed tissues of *V. faba*. *n* = 15 for apex, flower, stem and lower leaves, *n* = 14 for upper leaves. Significant differences among tissues are displayed by an asterisk.

Differences in PFAS accumulation between the different plant tissues were investigated for PFBA, PFPeA, PFHxA, PFOA, PFDA, and PFDoDA. Concentrations of PFPeA and PFHxA were significantly higher in the upper and lower leaves compared to the apex (*p* < 0.05, Figure 3b,c). PFHxA concentrations in the lower leaves were also higher than those in the flowers (*p* < 0.05, Figure 3c). The flowers contained higher PFOA concentrations than the other tissues (*p* < 0.05, Figure 3d). No differences were observed for PFBA, PFDA, and PFDoDA.

**Figure 4:**
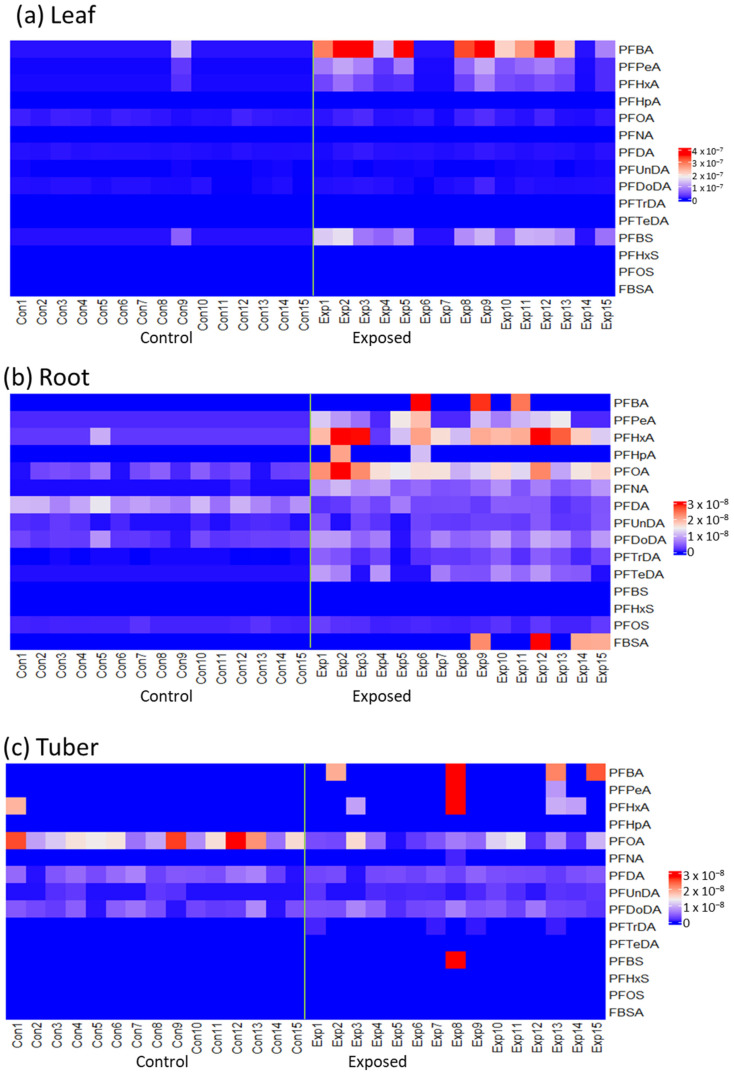
Heatmaps representing the relative PFAS concentrations in *R. raphanistrum* in mmol/g dw. The highest PFAS concentrations are shown in red, whereas the lowest concentrations are shown in blue. (**a**) concentrations in leaves; (**b**) concentrations in root; (**c**) concentrations in tubers. Con = control, Exp = exposed. *n* = 15 per treatment. The green line in each heatmap separates the control (Con) and exposed (Exp) plants on the x-axis.

In *R. raphanistrum* all PFASs, except for PFHxS, were regularly detected with PFBA being dominantly present in leaves and tubers, and PFOA being dominant in the roots (Figure 4, Table S5 for details).

**Figure 5:**
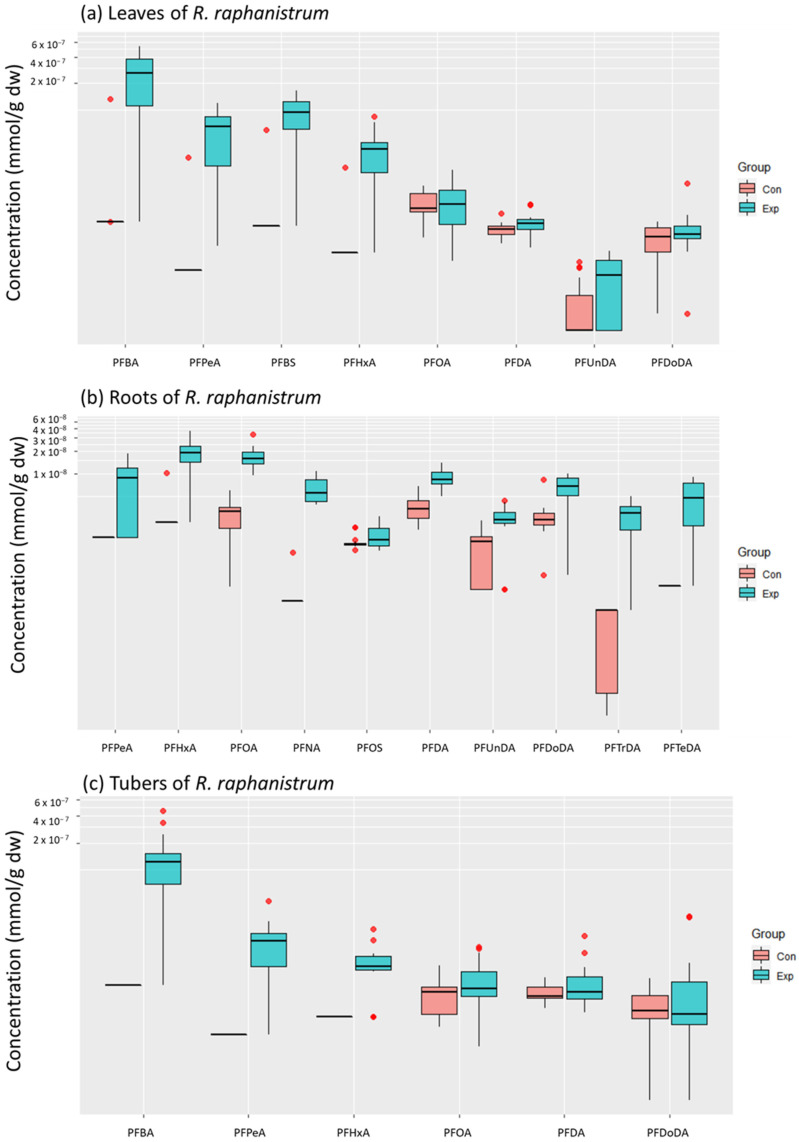
PFAS concentrations (mmol/g dw) in (**a**) leaves, (**b**) roots, and (**c**) tubers of exposed (Exp, blue) and control (Con, red) plants of *R. raphanistrum*. Outliers are indicated as red data points. Only compounds detected in at least 2/3 of the samples were included. *n* = 15 per treatment.

Similarly, as for *V. faba*, the PFAS concentrations in the leaves tended to decrease with increasing chain length, and significant differences between exposed and control plants were observed only for the short-chained PFBA, PFPeA, PFHxA, PFBS, and the long-chained PFUnDA (*p* < 0.05, Figure 5). In the roots, long-chained PFASs were more abundant, and significant differences between treatments (*p* < 0.05, Figure 5) were mainly observed for the long-chained PFOA, PFNA, PFDA, PFUnDA, PFTrDA, and PFTeDA, as well as for the short-chained PFHxA.

**Figure 6:**
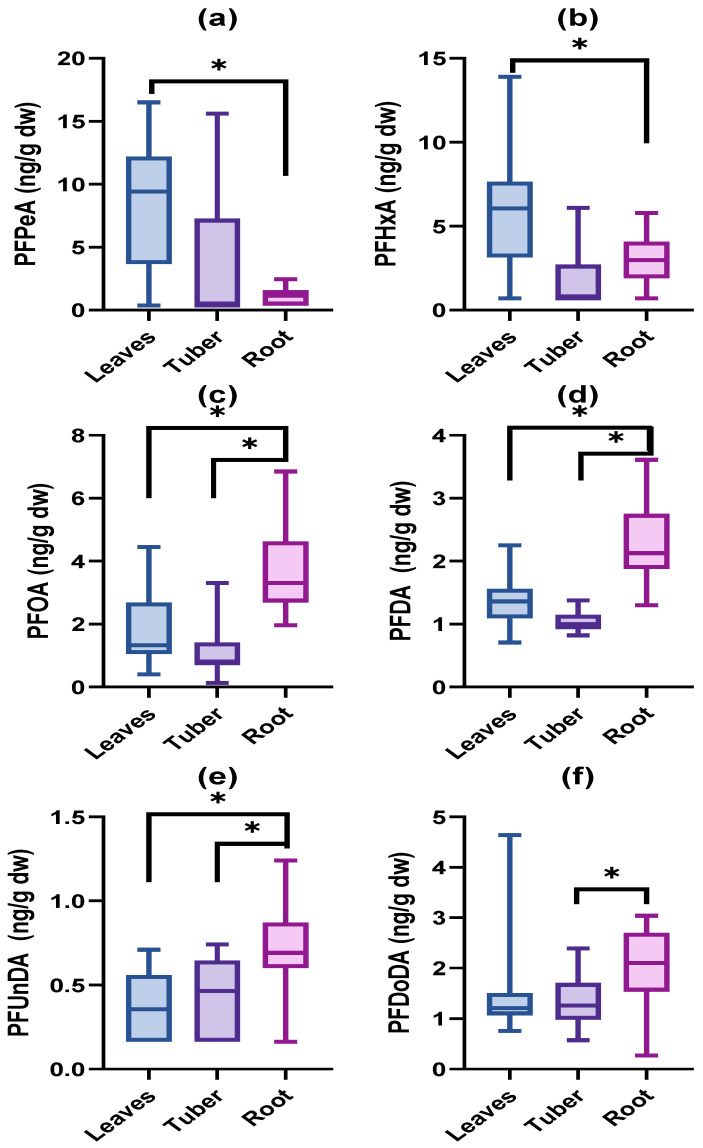
Concentrations (ng/g dw) of (**a**) PFPeA, (**b**) PFHxA, (**c**) PFOA, (**d**) PFDA, (**e**) PFUnDA, and (**f**) PFDoDA in leaves, tubers, and roots of *R. raphanistrum* exposed to spiked soils. Significant differences among tissues are displayed by an asterisk. *n* = 15 per tissue.

Concentrations of PFOA, PFDA, and PFUnDA were highest in the roots (*p* < 0.05; Figure 6c–e), followed by the leaves and the tubers, which did not differ from each other for neither of the three PFASs. PFDoDA concentrations were significantly (*p* < 0.05; Figure 6f) higher in the roots than in the tubers, but no other differences among tissues were observed. PFPeA and PFHxA concentrations could only be statistically compared between leaves and roots due to the low detection in the tubers. The concentrations of both PFASs were significantly (*p* < 0.05, Figure 6a,b) higher in the leaves than in the roots.

The translocation factor (TF, i.e., shoot/root concentration ratio) was calculated for each PFAS in *R. raphanistrum* to estimate the quotient of PFASs from roots to shoots (Table S6). All aboveground tissues were considered to be the shoot. The TF of PFPeA (6.94) was higher than that of other PFASs. In particular, the long-chained PFASs showed lower TFs (<1). The TFs showed the following order: PFPeA > PFHxA > PFDoDA > PFDA > PFOA > PFUnDA. Due to the low detection, no TFs could be determined for the other PFASs.

### 3.2. Growth Hormone Levels and Plant Growth

Only hormones that were detected with a frequency of at least 80% in all tissues were included in the association analyses. These included the cytokinins isopentenyl adenine (iPA) and *trans*-zeatin riboside (*t*ZR), the ethylene precursors ACC and its conjugates, IAA and its conjugates, JA, multiple GAs (GA1, GA7, GA8, GA12, GA15, GA19, GA20), MET, and ABA. The hormone levels ranged from <LOQ to 7.44 × 10^4^ pmol/g dw (Table S7 for *V. faba* and Table S8 for *R. raphanistrum*). In general, the hormone levels did not differ significantly between exposed and control plants for both species. The only exception was for MET levels in the apex of *V. faba* (Figure S1), which were significantly lower in the exposed plants compared to the control plants. 

**Figure 7:**
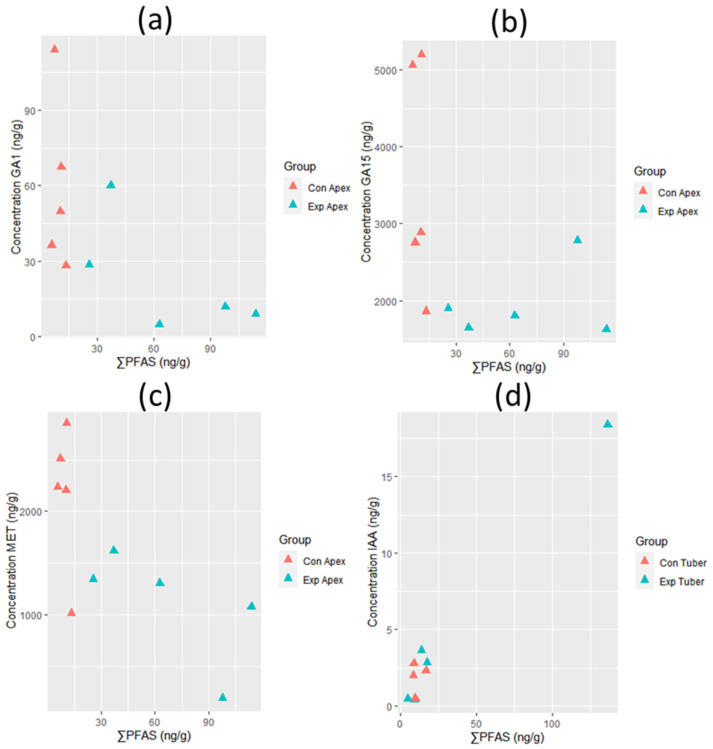
Correlations between hormone levels (ng/g dw) and ∑PFAS concentrations (ng/g dw) in the apex of *V. faba* ((**a**) (GA1, *n* = 10), (**b**) (GA15, *n* = 10), and (**c**) (MET, *n* = 10)) and tuber of *R. raphanistrum* ((**d**) (IAA, *n* = 8)). Controls are shown in red, whereas exposed plants are shown in blue.

The ∑PFAS concentrations in the apex of *V. faba* were significantly correlated to levels of GA1 (*p* = 0.03, rho = −0.71; Figure 7a) and GA15 (p = 0.04, rho = −0.68; Figure 7b). Furthermore, negative correlations were observed between ∑PFAS concentrations and MET levels in the apex of *V. faba* (p = 0.02, rho = −0.76; Figure 7c). The ∑PFAS concentrations in the tubers of *R. raphanistrum* were significantly correlated with the IAA levels (*p* = 0.08, rho = 0.63; Figure 7d). No other significant correlations with plant hormone levels were observed.

## 4. Discussion

The dominance of short-chained PFASs in aboveground tissues and the dominance of long-chained PFASs in roots is in agreement with findings in other studies (as has been reviewed by Ghisi et al. [12], Adu et al. [18], and Kavusi et al. [19]). PFAS uptake occurs primarily through energy-dependent active transport processes [20,21,22], which could involve carrier-mediated diffusion via aquaporins or transportation via anion channels [21,22]. Müller et al. [23] proposed a two-compartment uptake process for PFBA, which may involve both uptake by the root symplast and uptake by the central vasculature and other organelles. This process could be affected by active transport from the roots. Since transportation of nutrients to the aboveground tissues is primarily driven by transpiration [24], short-chained PFASs typically end up in the aboveground tissues due to their low vapor pressures and hydrophilicity [25,26].

*Vicia faba* is known to contain a heavily suberized exodermis, which might explain why PFAS concentrations in aboveground tissues tend to decrease with increasing carbon-chain length in this species in the present study. It was expected that long-chained PFASs would be more abundant in the aboveground tissues of *R. raphanistrum* since these plants lack Casparian strips between the tuber and aboveground shoot [9]. However, similar to *V. faba*, we found PFAS concentrations in the leaves to decrease with increasing chain length. This finding was also supported by translocation factors below 1 for the long-chain PFASs in *R. raphanistrum*. The Casparian strip is a hydrophobic layer of lignin and suberin-containing cells that constrains the translocation of PFASs from roots to the central vasculature of plants by blocking the nonliving spaces between cells and cell membranes [27,28]. In the exodermis of mature roots, the barrier to the apoplastic inflow of ions occurs near the root surface [29]. As a result, passive transport of (particularly hydrophobic) compounds across the exodermis is prevented in plants containing exodermal Casparian strips [30].

Besides the presence or absence of Casparian strips, the hydrophobicity and anion exchange characteristics of PFASs could affect their translocation within plants [31]. Plotting the TF against the carbon-chain length typically results in U-shaped patterns in which TFs are highest in short-chained hydrophilic PFASs, decrease for moderately hydrophobic PFASs, and then increase again for the most hydrophobic long-chained PFASs [23,32,33,34]. Although we could not determine TFs for all PFASs in the present study, our results show a similar pattern, with TFs being highest for PFPeA, followed by PFDoDA, and then the moderately hydrophobic PFASs with intermediate chain lengths.

PFAS uptake can induce metabolic responses in plants [13,18,35], including the production of metabolites or changes in enzymatic activity that may lead to phytotoxic effects of PFASs in plants [18,35]. To the best of our knowledge, no data is available about the possible effects of PFASs on plant hormone levels. Gibberellins are a large group of hormones that may regulate plant growth and development throughout the plant’s life cycle [36]. Lower levels of GA1 and GA15 due to PFAS exposure may reduce the size of the apical dome, hence affecting cell division [37,38], leading to smaller-sized plants. In addition, lower levels of these gibberellins may reduce stem elongation [39,40]. Methionine is involved in many processes, including the translation of proteins, the biosynthesis of ethylene, polyamines, and biotin, and the methylation of DNA [41,42]. A reduction of MET through PFAS exposure may thus lead to a variety of effects because of its fundamental role in plant cells. Since ethylene is involved in the senescence process [43], a reduction in MET levels may affect the aging process in *V. faba*. Despite the importance of IAA in cell division and elongation, as the main auxin in plants [44], IAA inhibits primary root elongation at high concentrations [45]. The positive association between PFAS concentrations and IAA levels in tubers of *R. raphanistrum* suggests that growth impairment may occur in this species, although this was not clearly seen in the plants.

This study shows that PFAS bioaccumulation in plants is both compound-specific, which is particularly affected by the carbon-chain length of the PFAS, as well as species-specific. Within species, PFASs tend to accumulate in different tissues, depending on their size and hydrophobicity, which is in agreement with the literature on PFAS bioaccumulation in plants. Furthermore, we provided the first evidence of associations between ∑PFAS accumulation in plants and their growth hormone levels. Despite the limited sample size in the present study, our results suggest that PFAS exposure to environmentally relevant concentrations may lead to growth reduction by affecting levels of methionine, gibberellins, and IAA in plants. In addition, PFAS exposure could affect the cell cycle in pericycle cells and methionine metabolism in plants. Given the complexity of plant hormone pathways, future studies should investigate the potential mechanisms of PFASs affecting plant hormone levels. Overall, as plant growth is a major component of plant fitness, an important mediator of competitive plant interactions, and a determinant of crop yield, PFAS exposure to plants may be both an ecological and economic concern.

## Data Availability

The datasets generated and/or analyzed during the current study are not publicly available. The test data is restricted to the relevant personnel of the project and is not allowed to be disclosed to the public but are available from the corresponding author on reasonable request.

## Supplementary Materials

The following supporting information can be downloaded at: https://www.mdpi.com/article/10.3390/toxics11110922/s1, Figure S1: Comparison of MET levels (ng/g dw) in the apex of exposed (blue) and control (red) plants of *V. faba* (*n* = 5 per treatment); Table S1: Mean PFAS concentration (± standard deviation) in ng/g dw in the spiked soil, *n* = 3; Table S2: Limits of quantification (LOQ) of the individual PFAS in plant matrices (ng/g dw); Table S3: MRM transitions, internal standards (ISTDs), cone voltages (V), and collision energy (eV) for the target perfluoroalkyl substances and their internal standards. Table adopted from Groffen et al. [46]; Table S4: PFAS concentrations (ng/g dw) and detection frequency (%) in the various tissues of exposed and control plants of *V. faba*. Values below LOQ are indicated as <LOQ. Ranges show minimum and maximum concentrations; Table S5: PFAS concentrations (ng/g dw) and detection frequency (%) in the various tissues of exposed and control plants of *R. raphanistrum*. Values below LOQ are indicated as <LOQ. Ranges show minimum and maximum concentrations; Table S6: Translocation factors of PFAS in exposed *R. raphanistrum* (*n* = 15); Table S7: Plant hormone levels (pmol/g dw) and detection frequency (%) in the upper leaves and apex of exposed and control plants of *V. faba*. Values below LOQ are indicated as <LOQ. Ranges show minimum and maximum concentrations. *n* = 5 per tissue for each of the treatments; Table S8: Plant hormone levels (pmol/g dw) and detection frequency (%) in the tubers and leaves of exposed and control plants of *R. raphanistrum*. Values below LOQ are indicated as <LOQ. Ranges show minimum and maximum concentrations. *n* = 5 per tissue for each of the treatments.

## Appendix A

PFAS extraction

To the homogenized samples, 10 ng of a mass-labeled internal standard (ISTD, MPFAC-MXA, Wellington Laboratories, Guelph, Canada) and 10 mL of acetonitrile (ACN) were added. Hereafter, samples were sonicated (Branson 2510) for 3 × 10 min, with vortexing in between, followed by an overnight incubation on a shaking plate (135 rpm). After centrifugation (1037× *g*, 4 °C, 10 min, Eppendorf centrifuge 5804R), the supernatant was concentrated to a volume of 0.5 mL using a rotational vacuum concentrator (Martin Christ RVC-2-25, Osterode am Harz, Germany) at 30 °C. The extracts were added to an Eppendorf tube containing 0.1 mL of graphitized carbon powder (Supelclean Envi-Carb, Sigma-Aldrich, Belgium) that was preconditioned with 50 μL of glacial acetic acid. The empty PP tubes were rinsed twice with 250 μL of ACN, and these rinse liquids were added to the same Eppendorf tube. After vortex-mixing, the sample was centrifuged (9279.4× *g*, 4 °C, 10 min, Eppendorf centrifuge 5415R), and the supernatant was completely dried under vacuum. The samples were reconstituted with 200 μL of a 2% ammonium hydroxide solution (diluted in ACN) and filtered through an Ion Chromatography Acrodisc 13 mm syringe filter with 0.2 μm Supor polyethersulfone (PES) membrane into a PP auto-injector vial before chemical analysis.

Hormone extraction

After 15 min of sonication, the samples were extracted overnight at −20 °C in 10 mL of 80% methanol (MeOH, *v*/*v*) per 1 g of plant material. 700 pmol internal tracers [^2^H_4_]1-aminocyclopropanecarboxylic acid (^2^H_4_]-ACC) (OlchemIm, Olomouc, Cz. Rep.), 1000 pmol 2-amino[^2^H_3_]4-methylsulfanylbutanoic acid ([^2^H_3_]-Met) (Sigma Aldrich, St. Louis, MO, USA), 20 pmol d-GA mix ([^2^H_2_]-GA1, [^2^H_2_]-GA4, [^2^H_2_]-GA8, [^2^H_2_]-GA9, [^2^H_2_]-GA15, [^2^H_2_]-GA19, [^2^H_2_]-GA20, (OlchemIm, Olomouc, Cz. Rep.), 200 pmol 3-[^13^C_6_]Indole-3-Acetic Acid (Cambridge Isotope Laboratories Inc., Tewksbury, MA, USA), 200 pmol (S)-5-[^2^H_6_](1-hydroxy-2,6,6-trimethyl-4-oxocyclohex-2-en-1-yl)-3-methyl-(2Z, 4E)-pentadienoic acid (^2^H_6_]-ABA) (OlchemIm, Olomouc, Cz Rep.) and 200 pmol dihydrojasmonic acid (diHJA, OlchemIm, Olomouc, Cz. Rep.), 100 pmol d-CKI mix ([^2^H_3_]dihydrozeatin, [^2^H_3_]dihydrozeatin riboside, [^2^H_5_]*trans*-zeatin-7-glucoside, [^2^H_5_]*trans*-zeatin-9-glucoside, [^2^H_6_]N6-isopentenyladenine, [^2^H_6_]*trans*-zeatin-*O*-glucoside, [^2^H_6_]*trans*-zeatin-*O*-glucoside riboside, [^2^H_6_]N6-isopentenyladenosine, [^2^H_6_]N6-isopentenyladenine-7-glucoside, [^2^H_6_]N6-isopentenyladenine-9-glucoside, Olchemim Ltd., Olomouc, Cz. Rep.)), d-CKA mix (([^2^H_7_]N6-benzyladenine (D-BA), [^2^H_7_]N6-benzyladenosine (D-BAR), [^2^H_7_]N6-benzyladenine-9-glucoside (D-BA9G), [^15^N_4_]*meta*-topolin (^15^N-*m*T), [^15^N_4_]*ortho*-topolin (^15^N-*o*T), Olchemim Ltd.) were added to the samples. Afterwards, 1 mg of hydrophilic-lipophilic balance powder (HLB, Waters Corporation, Milford, CT, USA) was added, and the samples were vortex-mixed. After centrifugation at 20,913× *g* and 4 °C for 20 min (Eppendorf Centrifuge 5810R, Eppendorf Hamburg, Germany) and recuperation of the supernatant, the extraction procedure was split into four different parts: (1) the ACC- and cytokinin fraction, (2) the total ACC-fraction, (3) the IAA fraction (including GA, JA, and ABA), and (4) the total IAA fraction.

A total of 80 μL of supernatant was filtered (Chromafil®, AO-20/3 filter, nylon, pore size 0.20 μm, 3 mm, Macherey-Nagel, Düren, Germany), collected in LC-MS vials, and stored at 4 °C for determination of cytokinins and the ACC fraction.

A 200 μL fraction was added to a 1 mL Pierce derivatization vial containing 200 μL of 4M hydrogen chloride (HCl). These samples were flushed under anoxic conditions with a water-saturated nitrogen stream for 20 min and hydrolyzed for 90 min at 100 °C (Pierce-Reacti-Therm III). After the supernatant was cooled and vortex-mixed, the samples were dried under a nitrogen flow at 40 °C (Zymark TurboVap LV Evaporator; Marshall Scientific, Hampton, NH, USA). The residual of the dried samples was redissolved in 80 μL of 80% MeOH in new LC-MS vials and stored at 4 °C for the determination of the total ACC fraction.

A 200 μL fraction was diluted with 0.8 mM formic acid and purified by binding the compounds of interest on a Bond-Elut C18 solid-phase extraction (SPE) cartridge. The analytes of interest were eluted with diethyl ether and dried under a nitrogen stream at 36 °C (Zymark TurboVap LV Evaporator; Marshall Scientific, Hampton, HH, USA). The samples were redissolved in a 60 μL 1-Ethyl-3-(3′-dimethylaminopropyl)carbodiimide (EDAC) solution (5 mg/60 μL EDAC, Merck Life Science bv, Hoeilaart, Belgium) in 100% MeOH (Hypercover CHROMANORM®, VWR, Leuven, Belgium). The samples were derivatized at 38 °C for 1 h at 900 rpm (Eppendorf Thermomixer Compact; Eppendorf, Hamburg, Germany) and dried under a nitrogen flow. After drying, the samples were redissolved in 60 μL of 10% MeOH and collected in new LC-MS vials. The samples were then stored at 4 °C prior to analyses of the IAA-fraction.

Finally, a 200 μL fraction was added to a 1 mL Pierce derivatization vial containing 200 μL of 14M sodium hydroxide (NaOH) and flushed under anoxic conditions with a water-saturated nitrogen stream for 20 min and hydrolyzed for 3 h at 100 °C (Pierce-Reacti- Therm III). After alkaline hydrolysis, samples were cooled, diluted 20 times with distilled water, and acidified with 2.5 mL of 2M HCl. After reaching a pH of 3, the samples were purified by binding the compounds of interest on a Bond-Elut C18 SPE cartridge (Agilent, Santa Clara, CA, USA). The analytes of interest were eluted with diethyl ether and dried under a nitrogen stream at 36 °C (Zymark TurboVap LV Evaporator; Marshall Scientific, Hampton, HH, USA). Afterward, the samples were redissolved in a 60 μL EDAC solution (5 mg EDAC) dissolved in 1 mL of 100% MeOH. Subsequently, the samples were derivatized at 38 °C for 1 h at 900 rpm (Eppendorf Thermomixer Compact; Eppendorf, Hamburg, Germany) and dried under a continuous nitrogen flow. After dissolving in 60 μL of 10% MeOH, all samples were measured using UPLC-MS/MS (ACQUITY, TQD, Waters, Milford, MA, USA) in positive ion electrospray ionization mode.

UPLC-MS/MS analyses

UPLC-MS/MS with negative electrospray ionization mode was used to analyze the PFAS concentrations. An ACQUITY BEH C18 column (2.1 mm × 50 mm; 1.7 μm, Waters, Milford, MA, USA) was used to separate the analytes. Between the injector and the solvent mixer, an ACQUITY BEH C18 pre-column (2.1 mm × 30 mm; 1.7 μm, Waters, Milford, MA, USA) was inserted to retain any PFAS contamination from the system. The mobile phase gradient started at 65% of 0.1% formic acid in water, changed to 100% of 0.1% formic acid in ACN in 3.4 min, and returned to 65% of 0.1% formic acid in water at 4.7 min. The flow rate was set at 450 μL/min with an injection volume of 6 μL (partial loop). Targeted PFAS were quantified using multiple reaction monitoring (MRM) of two diagnostic transitions per analyte, as validated by Groffen et al. [46]. Further specifications are displayed in Table S3.

Plant hormones were analyzed using a BEH C18 analytical column (130 Å, 1.7 μm, 2.1 mm × 50 mm; Waters, Milford, MA, USA) coupled to a BEH C18 VanGuard pre-column (130 Å, 1.7 μm, 2.1 mm × 5 mm; Waters, Milford, MA, USA). The used solvents were (A) 0.1% (*v*/*v*) formic acid in water, (B) 0.1% (*v*/*v*) formic acid in ACN, (C) 90/10 (*v*/*v*) ammonium acetate (1 mM)/MeOH, and (D) 100% MeOH. The mobile phase gradient for compounds IAA-ABA-JA started at 92:8 A:B, changed after 0.8 min to 60:40 A:B for 5 min, followed by a 60:40 to 10:90 transition in 30 s, after which it returned to 92:8 A:B at 6.1 min. For these hormones, a constant flow rate of 0.42 μL/min was used with a column temperature of 42 °C. For ACC, a gradient with a flow rate of 0.3 μL/min and a column temperature of 50 °C was used, starting at a solvent ratio of 99.9:0.1 A:B. This ratio changed after 1 min to 1:99 A:B for 5.4 min. The gradient returned to 99.9:0.1 A:B at 6.5 min. The gradient for isoprenoid cytokinins started at 95:5 C:D; after 0.4 min, it changed to 76:24 C:D, where it remained for 3 min. It then changed to 75:25 C:D at 5 min, 5:95 C:D at 6 min, and returned to 95:5 C:D after 6.5 min. Those of the aromatic cytokinins started at 99.9:0.1 C:D, changed to 72:28 C:D for 5 min, followed by 0.1:99.9 C:D after 5.5 min and 1:99 C:D at 6 min, and returned to 99.9:0.1 after 6.1 min. For both isoprenoid cytokinins and aromatic cytokinins, the flow rate was 0.4 μL/min with a column temperature of 50 °C.
