# Supplementary material for: Growth Hormones in Broad Bean (Vicia faba L.) and Radish (Raphanus raphanistrum subsp. sativus L.) Are Associated with Accumulated Concentrations of Perfluoroalkyl Substances
Source: Toxics. 2023 Nov 11;11(11):922. doi: 10.3390/toxics11110922 (PMC10674852; doi:10.3390/toxics11110922)
Supplement: Supplementary file 1 [file toxics-11-00922-s001.zip › toxics-2662730-supplementary.pdf]

Growth Hormones in Broad Bean (*Vicia faba* L.) and Radish (*Raphanus raphanistrum* subsp. *sativus* L.) Are Associated with Accumulated Concentrations of Perfluoroalkyl Substances

Thimo Groffen [1,*], Niels Kuijper [1], Sevgi Oden [2], Tim Willems [2], Lieven Bervoets [1] and Els Prinsen [2]

[1] ECOSPHERE, Department of Biology, University of Antwerp, Groenenborgerlaan 171, 2020 Antwerp, Belgium; ng.kuijper@student.maastrichtuniversity.nl (N.K.); lieven.bervoets@uantwerpen.be (L.B.)

[2] Integrated Molecular Plant Physiology Research (IMPRes), Department of Biology, University of Antwerp, Groenenborgerlaan 171, 2020 Antwerp, Belgium; sevgi.oden@uantwerpen.be (S.O.); tim.willems@uantwerpen.be (T.W.); els.prinsen@uantwerpen.be (E.P.)

* Correspondence: thimo.groffen@uantwerpen.be; Tel.: +31-6-41-20-90-80

Supplementary materials

Table S1. Mean PFAS concentration (± standard deviation) in ng/g dw in the spiked soil. $n = 3$.

PFAS	Concentrations (ng/g dw)
PFBA	8.07 ± 0.598
PFPeA	7.29 ± 0.434
PFHxA	8.84 ± 0.721
PFHpA	10.5 ± 1.09
PFOA	8.24 ± 0.584
PFNA	7.70 ± 0.311
PFDA	5.91 ± 0.400
PFUnDA	6.63 ± 0.696
PFDoDA	7.54 ± 0.882
PFTrDA	6.87 ± 0.543
PFTeDA	6.49 ± 0.385
PFBS	6.77 ± 0.857
PFHxS	10.8 ± 1.23
PFOS	6.85 ± 0.426
FBSA	6.18 ± 0.577

Table S2. Limits of quantification (LOQ) of the individual PFAS in plant matrices (ng/g dw).

PFAS	LOQ (ng/g dw)
PFBA	1.26
PFPeA	0.432
PFHxA	0.792
PFHpA	0.625
PFOA	0.143
PFNA	0.106
PFDA	0.223
PFUnDA	0.182
PFDoDA	0.305
PFTrDA	0.114
PFTeDA	0.257
PFBS	1.55
PFHxS	1.90
PFOS	0.0631
FBSA	0.206

Table S3. MRM transitions, internal standards (ISTDs), cone voltages (V), and collision energy (eV) for the target perfluoroalkyl substances and their internal standards. Table adopted from Groffen et al. [46].

Compound	Precursor ion (m/z)	Product Ion (m/z)		Cone Voltage (V) for Diagnostic Transition 1	Cone Voltage (V) for Diagnostic Transition 2	Collision Energy (eV) for Diagnostic Transition 1	Collision Energy (eV) for Diagnostic Transition 2	Internal Standard (ISTD) Used for Quantification
		Diagnostic Product Ion 1	Diagnostic Product Ion 2					
PFBA	213	169	169	19	19	19	50	$^{13}C_4$-PFBA
PFPeA	263	219	219	15	15	10	45	$^{13}C_4$-PFBA
PFHxA	313	269	119	19	19	21	65	[1,2-$^{13}C_2$]PFHxA
PFHpA	363	319	169	24	24	40	30	[1,2-$^{13}C_2$]PFHxA
PFOA	413	369	169	22	22	13	60	[1,2,3,4-$^{13}C_4$]PFOA
PFNA	463	419	169	28	28	17	20	[1,2,3,4,5-$^{13}C_5$]PFNA
PFDA	513	469	219	25	25	29	29	[1,2-$^{13}C_2$]PFDA
PFUnDA	563	519	169	18	18	30	35	[1,2-$^{13}C_2$]PFUnDA
PFDoDA	613	569	319	22	22	21	30	[1,2-$^{13}C_2$]PFDoDA
PFTrDA	663	619	319	26	26	21	30	[1,2-$^{13}C_2$]PFDoDA
PFTeDA	713	669	169	28	28	21	21	[1,2-$^{13}C_2$]PFDoDA
PFBS	299	80	99	40	40	65	45	$^{18}O_2$-PFHxS
PFHxS	399	80	99	22	22	30	60	$^{18}O_2$-PFHxS
PFOS	499	80	99	60	60	58	58	[1,2,3,4-$^{13}C_4$]PFOS
FBSA	298	219	78	34	40	27	38	$^{13}C_4$-PFBA
$^{13}C_4$-PFBA	217	172	172	19	19	19	50	
[1,2-$^{13}C_2$]PFHxA	315	269	119	19	19	21	65	
[1,2,3,4-$^{13}C_4$]PFOA	417	372	172	22	22	13	60	
[1,2,3,4,5-$^{13}C_5$]PFNA	468	423	172	28	28	17	20	
[1,2-$^{13}C_2$]PFDA	515	470	220	25	25	29	29	
[1,2-$^{13}C_2$]PFUnDA	565	520	170	18	18	32	35	
[1,2-$^{13}C_2$]PFDoDA	615	570	320	22	22	21	30	
$^{18}O_2$-PFHxS	403	84	103	22	22	30	60	
[1,2,3,4-$^{13}C_4$]PFOS	503	80	99	60	60	58	58	

Table S4. PFAS concentrations (ng/g dw) and detection frequency (%) in the various tissues of exposed and control plants of *V. faba*. Values below LOQ are indicated as <LOQ. Ranges show minimum and maximum concentrations.

		Control			Exposed		
**Tissue**	**Compound**	**Median**	**Range**	**Detection Frequency**	**Median**	**Range**	**Detection Frequency**
Upper leaves (n = 13 for control and n = 14 for exposed)	PFBA	<LOQ	<LOQ–<LOQ	0	32.3	13.9–72.2	100
	PFPeA	<LOQ	<LOQ–<LOQ	0	8.88	3.95–12.6	100
	PFHxA	<LOQ	<LOQ–<LOQ	0	4.00	1.78–6.49	100
	PFHpA	<LOQ	<LOQ–<LOQ	0	<LOQ	<LOQ–2.38	7
	PFOA	1.23	0.333–2.00	100	1.84	0.880–3.21	100
	PFNA	<LOQ	<LOQ–0.224	8	<LOQ	<LOQ–<LOQ	0
	PFDA	1.07	0.752–1.62	100	1.46	0.687–2.36	100
	PFUnDA	0.501	<LOQ–0.688	85	<LOQ	<LOQ–0.770	36
	PFDoDA	1.38	<LOQ–1.82	85	1.72	<LOQ–4.64	86
	PFTrDA	<LOQ	<LOQ–0.431	15	<LOQ	<LOQ–<LOQ	0
	PFTeDA	<LOQ	<LOQ–1.21	8	<LOQ	<LOQ–3.37	14
	PFBS	<LOQ	<LOQ–<LOQ	0	17.9	3.96–39.3	100
	PFHxS	<LOQ	<LOQ–<LOQ	0	<LOQ	<LOQ–<LOQ	0
	PFOS	<LOQ	<LOQ–<LOQ	0	<LOQ	<LOQ–<LOQ	0
	FBSA	<LOQ	<LOQ–<LOQ	0	<LOQ	<LOQ–<LOQ	0
Lower leaves (n = 15 for both treatments)	PFBA	<LOQ	<LOQ–<LOQ	0	34.5	<LOQ–72.0	87
	PFPeA	<LOQ	<LOQ–<LOQ	0	7.68	<LOQ–19.5	93
	PFHxA	<LOQ	<LOQ–<LOQ	0	5.17	<LOQ–9.66	93
	PFHpA	<LOQ	<LOQ–<LOQ	0	<LOQ	<LOQ–3.31	33
	PFOA	1.64	1.11–6.85	100	1.18	0.571–3.54	100
	PFNA	<LOQ	<LOQ–<LOQ	0	<LOQ	<LOQ–<LOQ	0
	PFDA	1.26	<LOQ–2.54	93	1.35	<LOQ–2.51	93
	PFUnDA	<LOQ	<LOQ–0.774	33	<LOQ	<LOQ–0.786	27
	PFDoDA	1.50	<LOQ–6.03	63	1.36	<LOQ–2.35	87
	PFTrDA	<LOQ	<LOQ–<LOQ	0	<LOQ	<LOQ–0.381	7
	PFTeDA	<LOQ	<LOQ–<LOQ	0	<LOQ	<LOQ–<LOQ	0
	PFBS	<LOQ	<LOQ–<LOQ	0	40.6	<LOQ–69.7	93
	PFHxS	<LOQ	<LOQ–<LOQ	0	<LOQ	<LOQ–<LOQ	0
	PFOS	<LOQ	<LOQ–<LOQ	0	<LOQ	<LOQ–0.191	7
	FBSA	<LOQ	<LOQ–<LOQ	0	<LOQ	<LOQ–<LOQ	0
Stem (n = 15 for both treatments)	PFBA	<LOQ	<LOQ–<LOQ	0	<LOQ	<LOQ–22.0	53
	PFPeA	<LOQ	<LOQ–<LOQ	0	<LOQ	<LOQ–5.41	27
	PFHxA	<LOQ	<LOQ–1.78	13	<LOQ	<LOQ–2.40	47
	PFHpA	<LOQ	<LOQ–<LOQ	0	<LOQ	<LOQ–<LOQ	0
	PFOA	2.52	0.434–5.05	100	2.64	<LOQ–5.10	93
	PFNA	<LOQ	<LOQ–0.844	7	<LOQ	<LOQ–<LOQ	0
	PFDA	1.09	<LOQ–2.49	87	1.41	<LOQ–2.54	93
	PFUnDA	0.349	<LOQ–1.51	53	<LOQ	<LOQ–0.886	40
	PFDoDA	1.10	<LOQ–3.81	80	1.21	<LOQ–3.31	80
	PFTrDA	<LOQ	<LOQ–7.00	7	<LOQ	<LOQ–<LOQ	0
	PFTeDA	<LOQ	<LOQ–4.42	7	<LOQ	<LOQ–<LOQ	0
	PFBS	<LOQ	<LOQ–<LOQ	0	<LOQ	<LOQ–<LOQ	0
	PFHxS	<LOQ	<LOQ–<LOQ	0	<LOQ	<LOQ–<LOQ	0
	PFOS	<LOQ	<LOQ–<LOQ	0	<LOQ	<LOQ–<LOQ	0
	FBSA	<LOQ	<LOQ–<LOQ	0	<LOQ	<LOQ–<LOQ	0

Flower (*n* = 13 for control and *n* = 15 for exposed)	PFBA	<LOQ	<LOQ–<LOQ	0	17.8	4.07–78.5	100
	PFPeA	<LOQ	<LOQ–<LOQ	0	2.92	<LOQ–19.7	93
	PFHxA	<LOQ	<LOQ–9.25	7	2.26	<LOQ–10.8	87
	PFHpA	<LOQ	<LOQ–<LOQ	0	<LOQ	<LOQ–<LOQ	0
	PFOA	2.22	1.17–18.7	100	2.75	1.20–11.7	100
	PFNA	<LOQ	<LOQ–<LOQ	0	<LOQ	<LOQ–<LOQ	0
	PFDA	1.65	1.15–11.8	100	1.38	0.940–7.36	100
	PFUnDA	<LOQ	<LOQ–0.946	33	<LOQ	<LOQ–0.933	40
	PFDoDA	1.82	0.783–22.0	100	1.06	<LOQ–20.2	73
	PFTrDA	<LOQ	<LOQ–<LOQ	0	<LOQ	<LOQ–<LOQ	0
	PFTeDA	<LOQ	<LOQ–<LOQ	0	<LOQ	<LOQ–<LOQ	0
	PFBS	<LOQ	<LOQ–<LOQ	0	<LOQ	<LOQ–<LOQ	0
	PFHxS	<LOQ	<LOQ–<LOQ	0	<LOQ	<LOQ–<LOQ	0
	PFOS	<LOQ	<LOQ–<LOQ	0	<LOQ	<LOQ–<LOQ	0
	FBSA	<LOQ	<LOQ–<LOQ	0	<LOQ	<LOQ–<LOQ	0
Apex (*n* = 15 for both treatments)	PFBA	<LOQ	<LOQ–<LOQ	0	16.4	<LOQ–61.4	93
	PFPeA	<LOQ	<LOQ–<LOQ	0	2.67	<LOQ–7.48	93
	PFHxA	<LOQ	<LOQ–<LOQ	0	1.67	<LOQ–4.34	87
	PFHpA	<LOQ	<LOQ–<LOQ	0	<LOQ	<LOQ–<LOQ	0
	PFOA	1.05	0.425–2.08	100	1.32	0.736–3.62	100
	PFNA	<LOQ	<LOQ–<LOQ	0	<LOQ	<LOQ–<LOQ	0
	PFDA	1.17	0.864–1.89	100	1.40	0.837–6.00	100
	PFUnDA	0.369	<LOQ–0.771	53	<LOQ	<LOQ–0.567	47
	PFDoDA	0.957	<LOQ–2.24	80	1.12	<LOQ–11.7	80
	PFTrDA	<LOQ	<LOQ–<LOQ	0	<LOQ	<LOQ–<LOQ	0
	PFTeDA	<LOQ	<LOQ–0.798	7	<LOQ	<LOQ–<LOQ	0
	PFBS	<LOQ	<LOQ–<LOQ	0	<LOQ	<LOQ–<LOQ	0
	PFHxS	<LOQ	<LOQ–<LOQ	0	<LOQ	<LOQ–<LOQ	0
	PFOS	<LOQ	<LOQ–<LOQ	0	<LOQ	<LOQ–<LOQ	0
	FBSA	<LOQ	<LOQ–<LOQ	0	<LOQ	<LOQ–2.32	13

Table S5. PFAS concentrations (ng/g dw) and detection frequency (%) in the various tissues of exposed and control plants of *R. raphanistrum*. Values below LOQ are indicated as <LOQ. Ranges show minimum and maximum concentrations.

		Control			Exposed		
Tissue	Compound	Median	Range	Detection Frequency	Median	Range	Detection Frequency
	PFBA	<LOQ	<LOQ–16.1	7	32.5	<LOQ–59.7	87
	PFPeA	<LOQ	<LOQ–4.23	7	9.42	<LOQ–16.5	87
	PFHxA	<LOQ	<LOQ–3.38	7	6.07	<LOQ–13.9	87
	PFHpA	<LOQ	<LOQ–<LOQ	0	<LOQ	<LOQ–<LOQ	0
	PFOA	1.73	<LOQ–3.12	93	1.33	0.400–4.45	100
Leaf	PFNA	<LOQ	<LOQ–<LOQ	0	<LOQ	<LOQ–<LOQ	0
(*n* = 15	PFDA	1.24	0.579–1.89	100	1.36	0.710–2.25	100
for	PFUnDA	<LOQ	<LOQ–0.544	40	0.357	<LOQ–0.709	60
both	PFDoDA	1.11	<LOQ–1.72	80	1.21	0.758–4.64	100
treatments)	PFTrDA	<LOQ	<LOQ–0.316	7	<LOQ	<LOQ–<LOQ	0
	PFTeDA	<LOQ	<LOQ–1.63	7	<LOQ	<LOQ–<LOQ	0
	PFBS	<LOQ	<LOQ–8.72	7	15.5	<LOQ–26.2	87
	PFHxS	<LOQ	<LOQ–<LOQ	0	<LOQ	<LOQ–<LOQ	0
	PFOS	<LOQ	<LOQ–0.168	7	<LOQ	<LOQ–<LOQ	0
	FBSA	<LOQ	<LOQ–<LOQ	0	<LOQ	<LOQ–<LOQ	0
	PFBA	<LOQ	<LOQ–<LOQ	0	3.15	<LOQ–37.6	47
	PFPeA	<LOQ	<LOQ–<LOQ	0	<LOQ	<LOQ–15.6	33
	PFHxA	<LOQ	<LOQ–2.85	7	<LOQ	<LOQ–6.09	40
	PFHpA	<LOQ	<LOQ–<LOQ	0	<LOQ	<LOQ–<LOQ	0
	PFOA	3.00	1.14–6.17	100	0.817	<LOQ–3.31	93
Tuber	PFNA	<LOQ	<LOQ–<LOQ	0	<LOQ	<LOQ–0.466	27
(*n* = 15	PFDA	0.984	<LOQ–1.71	87	1.00	0.821–1.38	100
for	PFUnDA	<LOQ	<LOQ–0.610	27	0.465	<LOQ–0.740	67
both	PFDoDA	0.914	<LOQ–1.74	80	1.26	0.573–2.39	100
treatments)	PFTrDA	<LOQ	<LOQ–<LOQ	0	<LOQ	<LOQ–0.979	27
	PFTeDA	<LOQ	<LOQ–<LOQ	0	<LOQ	<LOQ–<LOQ	0
	PFBS	<LOQ	<LOQ–<LOQ	0	<LOQ	<LOQ–27.3	33
	PFHxS	<LOQ	<LOQ–<LOQ	0	<LOQ	<LOQ–<LOQ	0
	PFOS	<LOQ	<LOQ–<LOQ	0	<LOQ	<LOQ–<LOQ	0
	FBSA	<LOQ	<LOQ–<LOQ	0	<LOQ	<LOQ–<LOQ	0
	PFBA	<LOQ	<LOQ–<LOQ	0	<LOQ	<LOQ–4.92	20
	PFPeA	<LOQ	<LOQ–<LOQ	0	1.15	<LOQ–2.45	67
	PFHxA	<LOQ	<LOQ–1.91	7	2.98	<LOQ–5.79	93
	PFHpA	<LOQ	<LOQ–<LOQ	0	<LOQ	<LOQ–3.79	13
	PFOA	0.774	<LOQ–1.50	80	3.31	1.96–6.85	100
Root	PFNA	<LOQ	<LOQ–0.250	7	1.29	0.894–2.52	100
(*n* = 15	PFDA	1.05	0.549–2.08	100	2.13	1.30–3.61	100
for	PFUnDA	0.425	<LOQ–0.808	60	0.687	<LOQ–1.24	87
both	PFDoDA	0.907	<LOQ–3.10	93	2.10	<LOQ–3.04	93
treatments)	PFTrDA	<LOQ	<LOQ–0.663	47	0.991	<LOQ–1.65	93
	PFTeDA	<LOQ	<LOQ–<LOQ	0	1.70	<LOQ–3.24	73
	PFBS	<LOQ	<LOQ–<LOQ	0	<LOQ	<LOQ–<LOQ	0
	PFHxS	<LOQ	<LOQ–<LOQ	0	<LOQ	<LOQ–<LOQ	0
	PFOS	<LOQ	<LOQ–0.583	40	0.327	<LOQ–0.679	80
	FBSA	<LOQ	<LOQ–<LOQ	0	<LOQ	<LOQ–4.44	27

Table S6. Translocation factors of PFAS in exposed *R. raphanistrum* (n = 15).

PFAS	Translocation Factor
PFPeA	6.94
PFHxA	1.68
PFOA	0.51
PFDA	0.56
PFUnDA	0.46
PFDoDA	0.67

Table S7. Plant hormone levels (pmol/g dw) and detection frequency (%) in the upper leaves and apex of exposed and control plants of *V. faba*. Values below LOQ are indicated as <LOQ. Ranges show minimum and maximum concentrations. n = 5 per tissue for each of the treatments.

Tissue	Compound		Control			Exposed		
			Median	Range	Detection Frequency	Median	Range	Detection Frequency
Upper leaf	Cytokinines	Trans-ZR	<LOQ	<LOQ–39.3	40	<LOQ	<LOQ–15.3	40
		iPA	0.891	<LOQ–9.85	80	1.07	<LOQ–8.59	80
	ACC	ACC	28.8	26.4–39.0	100	27.8	19.0–46.9	100
		ACC-Conjugates	39.7	<LOQ–132	80	55.3	46.8–288	100
	IAA	IAA	34.6	14.1–90.9	100	17.1	10.6–43.9	100
		IAA-Conjugates	109	11.5–4029	100	82.3	<LOQ–95.9	80
	JA	JA	82.5	<LOQ–404	60	70.1	<LOQ–333	80
	Gibberellins	GA1	24.9	6.83–68.8	100	33.1	11.2–103	100
		GA7	1248	679–3155	100	463	302–1936	100
		GA8	31.0	<LOQ–110	60	<LOQ	<LOQ–39.4	40
		GA12	1490	13.0–10,238	100	1254	7.96–9877	100
		GA15	1229	542–1425	100	666	534–1027	100
		GA19	62.0	20.8–186	100	68.1	46.3–113	100
		GA20	<LOQ	<LOQ–37.1	40	<LOQ	<LOQ–29.5	40
	MET	MET	446	231–840	100	298	211–651	100
	ABA	ABA	123	<LOQ–217	60	204	48.3–397	100
Apex	Cytokinines	Trans-ZR	207	<LOQ–437	80	286	<LOQ–1130	80
		IPA	167	2.29–976	100	81.9	1.56–243	100
	ACC	ACC	52.9	19.0–61.3	100	44.0	30.6–86.7	100
		Conjugates	60.1	<LOQ–190	80	51.8	41.9–107	100
	IAA	IAA	90.7	52.1–111	100	95.6	34.8–143	100
		Conjugates	219	<LOQ–405	80	19.4	<LOQ–495	60
	JA	JA	60.2	<LOQ–164	80	5.48	0.465–80.3	100
	Gibberellins	GA1	49.6	28.2–114	100	11.9	4.89–60.1	100
		GA7	3018	1426–19,217	100	3123	1826–12,204	100
		GA8	73.4	<LOQ–662	80	137	<LOQ–190	80
		GA12	13,793	13.8–270,149	100	128,498	92.1–305,121	100
		GA15	2887	1863–5197	100	1804	1631–2783	100
		GA19	25.9	<LOQ–255	80	77.8	61.0–115	100
		GA20	66.7	41.1–875	100	94.2	25.7–182	100
	MET	MET	2235	1010–2856	100	1301	193–1619	100
	ABA	ABA	80.6	37.7–971	100	896	53.3–6643	100

Table S8. Plant hormone levels (pmol/g dw) and detection frequency (%) in the tubers and leaves of exposed and control plants of *R. raphanistrum*. Values below LOQ are indicated as <LOQ. Ranges show minimum and maximum concentrations. n = 5 per tissue for each of the treatments.

Tissue	Compound		Control Median	Control Range	Control Detection Frequency	Exposed Median	Exposed Range	Exposed Detection Frequency
Tuber	Cytokinines	Trans-ZR	<LOQ	<LOQ–5.83	40	1.47	<LOQ–9.76	60
		IPA	5.94	<LOQ–39.2	80	6.65	<LOQ–37.3	80
	ACC	ACC	90.4	<LOQ–365	80	816	90.3–1208	100
		Conjugates	283	<LOQ–915	60	816	<LOQ–1208	80
	IAA	IAA	10.8	7.89–34.4	100	73.7	10.1–3245	100
		Conjugates	0.0234	<LOQ–236	60	110	<LOQ–281	80
	JA	JA	<LOQ	<LOQ–0.0889	20	38.2	0.115–464	100
	Gibberellins	GA1	10.2	7.53–43.2	100	12.9	<LOQ–37.4	60
		GA7	257	172–463	100	255	<LOQ–411	80
		GA8	<LOQ	<LOQ–<LOQ	0	<LOQ	<LOQ - <LOQ	0
		GA12	<LOQ	<LOQ–12.5	40	<LOQ	<LOQ–2621	20
		GA15	798	449–3113	100	1308	<LOQ–3203	80
		GA19	<LOQ	<LOQ–6.72	20	<LOQ	<LOQ–<LOQ	0
		GA20	9.86	<LOQ–14.3	80	10.9	<LOQ–16.9	80
	MET	MET	346	165–772	100	714	235–1562	100
	ABA	ABA	107	75.3–594	100	96.4	<LOQ–544	80
Leaves	Cytokinines	Trans-ZR	<LOQ	<LOQ–<LOQ	0	<LOQ	<LOQ–11.1	20
		IPA	<LOQ	<LOQ–5.21	40	<LOQ	<LOQ–2.65	40
	ACC	ACC	145	<LOQ–564	80	245	89.5–387	100
		Conjugates	244	<LOQ–680	60	379	<LOQ–571	80
	IAA	IAA	49.1	21.3–69.3	100	66.5	18.4–7606	100
		Conjugates	5196	<LOQ–74,403	80	2512	<LOQ–5218	80
	JA	JA	0.189	<LOQ–161	60	1.05	<LOQ–2405	80
	Gibberellins	GA1	18.0	<LOQ–99.7	80	11.3	<LOQ–133	60
		GA7	570	384–1472	100	254	<LOQ–419	80
		GA8	41.3	<LOQ–508	60	<LOQ	<LOQ–1396	20
		GA12	5.27	<LOQ–668	60	16.8	<LOQ–1666	60
		GA15	645	497–3337	100	446	<LOQ–3614	80
		GA19	<LOQ	<LOQ–11.4	40	<LOQ	<LOQ–10.4	40
		GA20	22.5	15.9–76.7	100	27.6	<LOQ–40.0	80
	MET	MET	314	245–539	100	347	166–775	100
	ABA	ABA	42.2	27.4–339	100	39.7	<LOQ–203	80

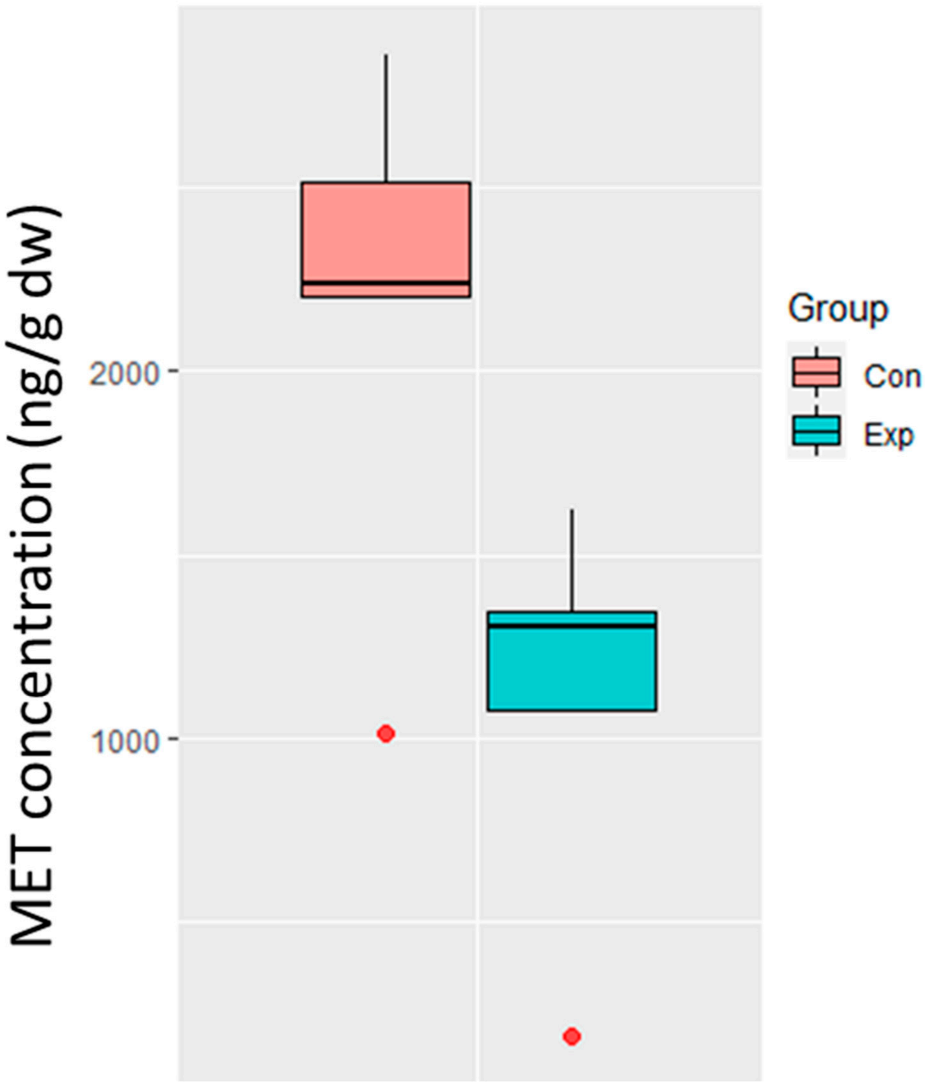

Figure S1. Comparison of methionine (MET) levels (ng/g dw) in the apex of exposed (blue) and control (red) plants of *V. faba* (n = 5 per treatment).
